# Adaptive Pressure Control System Based on the Maximum Correntropy Criterion

**DOI:** 10.3390/s21155156

**Published:** 2021-07-30

**Authors:** Thommas Kevin Sales Flores, Juan Moises Mauricio Villanueva, Heber Pimentel Gomes, Sebastian Yuri Cavalcanti Catunda

**Affiliations:** 1 Renewable and Alternatives Energies Center (CEAR), Electrical Engineering Department (DEE), Campus I, Federal University of Paraiba (UFPB), Joao Pessoa 5045, PB, Brazil; jmauricio@cear.ufpb.br; 2 Technology Center (CT), Department of Civil and Environmental Engineering (DECV), Campus I, Federal University of Paraiba (UFPB), Joao Pessoa 5045, PB, Brazil; heberp@uol.com.br; 3 Computer and Automation Engineering Department (DCA), Federal University of Rio Grande do Norte, Natal 59078-970, RN, Brazil; catundaz@gmail.com

**Keywords:** correntropy, adaptive system, water pumping

## Abstract

Water supply systems are constantly improving their operation through energy efficiency actions that involve the use of advanced measurement, control, and automation techniques. The maintenance and reliability of water distribution is directly associated with hydraulic pressure control. The main challenges encountered in hydraulic pressure control are associated with random changes in the supply plant and the presence of noise and outliers in the sensor measurements. These undesired characteristics cause inefficiency and instability in the control system of the pumping stations. In this scenario, this paper proposes an indirect adaptive control methodology by reference model for modeling and controlling water supply systems. The criterion adopted in the parametric estimation mechanism and the controller adaptation is the Maximum Correntropy. Experimental results obtained with an experimental bench plant showed that the maximum tracking error was 15% during demand variation, percentage overshoot less than 5%, and steady-state error less than 2%, and the control system became robust to noise and outliers. In comparison to the Mean Squared Error criterion, when noise and outliers influence the sensor signal, the proposed methodology stands out, reducing the mean error and the standard deviation, in the worst-case scenario, by more than 1500%. The proposed methodology, therefore, allows for increased reliability and efficiency of an advanced pump control system, avoiding downtime and equipment damage.

## 1. Introduction

The economy, reliability, and stability of water supply systems have a direct impact on the various sectors of society, and to improve these characteristics, the utility companies continuously invest in infrastructure, monitoring, and new information technology instruments, transforming the water supply networks into an intelligent system [1].

In the modern water supply system, various techniques have been used to monitor and control the hydraulic pressure, trying to increase its reliability and continuity. Studies such as those developed by [2,3,4] have made comparisons between Proportional Integral Derivative (PID) control and PID-Fuzzy controllers. The results showed that Fuzzy control is more robust to variations in physical plant characteristics and noise in signals measurement, unlike PID control, whose gains are determined to meet a desired dynamic response.

In [5], an intelligent control system based on an Artificial Neural Network (ANN) with multilayer feedforward architecture was proposed for the operation of a water supply system with parallel pumps and with electric motors driven by a frequency converter. The settling time in all experiments was less than 30 Therefore, and the maximum relative steady-state error was 2.9%. In addition, the generalization capacity associated with the ANN algorithm allowed for an increase in the hydraulic and energy efficiency of the operations, a reduction in water losses, and an increase in the useful life of the equipment.

In [6], a Reference Model Adaptive Control (MRAC) system was carried out for a conical water tank system, showing its robustness and high performance to randomness, i.e., adapting the model parameters to the parametric variations of the system, such as roughness, temperature, water density, and others. An adaptive PID control by gain scaling was developed in [7], applied to pressure regulating valves in water distribution networks. In [8], a self-adjusting Adaptive Controller with Generalized Minimum Variance (GMV) was developed for the modeling and real-time control of a water pumping system. In this strategy, control valves were used, and a frequency converter was used to drive a motor-pump set simultaneously. In [9], a self-adjusting Adaptive Controller was proposed to manage, throughout the day, the hydraulic pressure at the nodes of a water distribution network by managing the water level in a storage tank. As a result, water supply was provided to different topographical zones of a city to meet the required demands.

Substantially, good quality in the estimation of measurements with low associated uncertainty is an important aspect to ensure the stability of these control systems. However, the measured values can be affected by other factors, such as communication errors, incorrect operations of transducers, and acquisition equipment failures. These atypical values are called outliers. Therefore, for the adaptive control system to perform appropriately, suitable integrity of the data is required; otherwise, the results may lead to instability, damaging the hydraulic structure [10,11].

Usually, in adaptive control systems, the Mean Squared Error (MSE) is used as the error minimization criterion to determine the model parameters. However, to use the MSE, one must consider that there is a Gaussian probability density function (PDF) of the error, with zero mean and known standard deviation, and that there is a linear correlation between the concerning variables [12,13]. However, in real engineering applications, the PDF of the error is unknown, so the MSE as a minimization criterion for the determination of the model parameters does not transfer all information contained in the dataset which are mainly the higher-order statistical moments (Skewness and Kurtosis) [14,15].

In this work, we propose an innovative methodology for adaptive pressure control in a hydraulic system, based on Indirect Model Reference Adaptive Control with Maximum Correntropy Criterion (IMRAC-MCC), to improve the operational performance of water pumping systems. The main contribution is the development of an adaptive control algorithm capable of naturally rejecting the influence of outliers, which uses fewer parameters for tuning than the MSE approach.

From the development of this work, we can highlight the main contributions:

(i) The development of an adaptive control algorithm capable of naturally rejecting the influence of outliers;
(ii) Control algorithm based on MCC with fewer parameters to be used for tuning than mean squared error (MSE);
(iii) Increased reliability in pumping systems, avoiding measurement problems or operational failures that cause instability in the pumping system and, consequently, the assignment of this service.

For the validation and comparison of the experimental results between the MSE and MCC minimization criteria, a fully automated experimental water pumping system was used, located in the Laboratory of Energy Efficiency and Hydraulics in Sanitation (LENHS) at the Federal University of Paraiba (UFPB).

## 2. Related Works

With population growth in large urban centers caused by verticalization and with the rapid increase of inner-city apartment high-rise buildings, there is also a need for more efficient techniques to provide water supply system reliability. Given this scenario, water and sanitation companies are investing in infrastructure, micro and macro measurement and advanced control systems. In the literature review, works directly related to this problem were found and will be briefly addressed below.

An Interactive Learning Control (ILC) controller was developed in [16] for pressure control in a water supply system. This proposed control provides pressure set-points for all inputs to the network instead of the flow rate, reducing the number of flow meters, which are typically more costly. In this way, the design of the controller does not depend on a model, but on the periodicity of the signal to be controlled.

In [17], was investigated the stability and robustness of Real-Time Control (RTC) algorithms based on models systems dynamic linear water supply around the operating point. The analysis concludes that the instability may be caused by multiple resonance conditions and the different system gains. Moreover, a low-pass filter and a Smith predictor (predictive controller) were used to improve control robustness.

In [9], a self-adjusting Adaptive Controller was proposed to manage, throughout the day, the hydraulic pressure at the nodes of a water distribution network by controlling the level in a water tank. The aim was to provide an equal supply for the demand required in different areas of a city. It was verified, from the results of three consecutive days of operation, that the linear model used performed the pressure estimation with an error smaller than 5% for each node of the network.

Refs. [18,19] presented a Predictive Model Controller for a large water distribution network. The proposed algorithms were used to optimize the water level in the reservoirs and the node pressure at each point. The control behaved as designed, ensuring the stability of the node pressure.

Proportional Integral (PI) control development was showed in [20], whose objective was to increase the energy efficiency and reliability of water pumping units with cascade pumps. The dynamic error for two cascade pumps did not exceed 3% and also reduced the energy losses per one-day cycle by 30%.

A non-convex Neuro-Linguistic Programming (NLP) was used in [21] to carry out mono-objective and multi-objective optimization of the control curves for the variable speed pumps and pressure reducing valves. The target function considered the mean zonal pressure, the energy consumption of the water pump, and the cost of water treatment. The controller proved to be robust to the uncertainty of the hydraulic system and the demand changes.

On the other hand, in [2] fuzzy logic was used to adjust a pressure control system and an experimental water supply network. During the experiments, three pumping system operation configurations were setting: series, parallel and single pump. The results showed that all configurations of the pressure control system were able to maintain stability, but of the three, the parallel operation presented the highest energy efficiency.

The controllers cited above, despite presenting satisfactory results, suffer when the measured signal is influenced by outliers. To circumvent this, Reference [22] proposes adaptive inverse control, based on the Maximum Correntropy Criterion (MCC) algorithm, to circumvent the limitations found in the least squares method (LMS), which works well only for linear and Gaussian systems. The MCC algorithm aims to maximize the correntropy between the model output and the desired response. Since correntropy is a non-linear similarity measure that contains higher order statistics of the signals and is insensitive to large discrepancy values, it is therefore possible to achieve desirable performance in impulsive noise environments [23,24].

In [25], a maximum recursive filtered-x correntropy (FxRMC) algorithm is proposed based on the Maximum Correntropy Criterion (MCC) to reduce the effect of outliers. The proposed FxRMC algorithm requires no previous information of the noise characteristics and outperforms the filtered x-filtered least mean square (FxLMS) algorithm for impulsive noise. Meanwhile, in order to adjust the kernel size of the online FxRMC algorithm, a recursive approach was adopted by taking into account the previous estimates of error signals over a sliding window.

According to the literature review, several current control and system identification strategies are currently being developed. However, these techniques are mainly based on building the adaptive models using the MSE criterion, that is, considering that the PDF of the error between the estimated value and the measured one is of Gaussian type and is correlated. To solve these limitations, it is necessary to conduct research that allows the development of adaptive systems capable of maximizing the extraction of information from the error PDF, considering that the system, in general, presents non-linear and time-varying characteristics.

## 3. Measuring Setup and Data Acquisition

**Figure 1:**
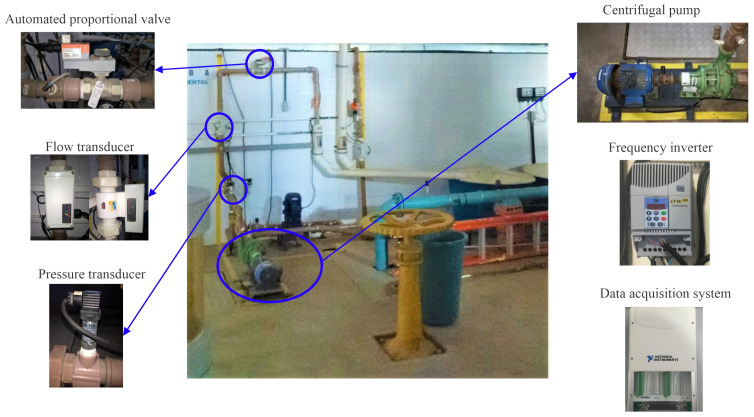
Photograph of the experimental system.

The main function of water supply systems is to provide the population with drinking water in appropriate quantity and pressure. The efficient management and operation of this type of system involves the application of control and automation strategies. In this work, an experimental system was used, as illustrated in Figure 1, which emulates a water supply system with variable demand. This experimental system is located in the Laboratory of Energy Efficiency and Hydraulics in Sanitation (LENHS) at the Federal University of Paraíba (UFPB) in João Pessoa, Brazil.

**Figure 2:**
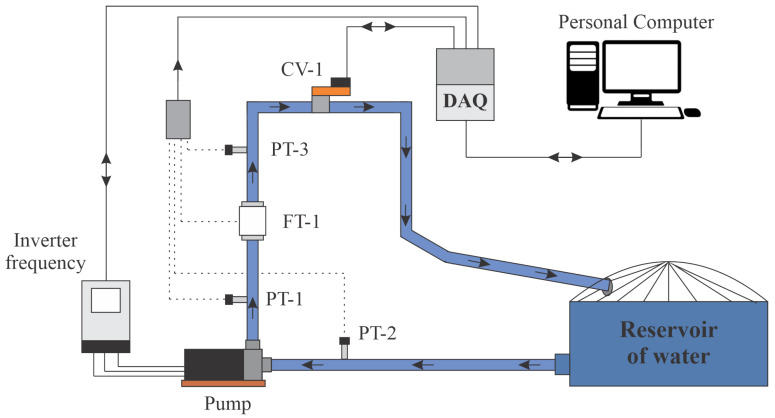
Schematic of the experimental system.

Figure 2 shows the schematic diagram of the experimental system, in which water from the reservoir is pumped by a centrifugal pump (three-phase 220/380 V 3 hp) through Polyvinyl chloride (PVC) pipes and connections. The pump supplies the liquid with energy in the form of pressure and flow, which are measured using pressure (PT) and flow (FT) transducers, whose maximum measurement limits are 42.21 m H${}_{2}$O and 11.34 L/s, respectively.

The pump’s rotation speed is controlled by a frequency converter. In addition, at the outlet of the system is an automated proportional valve (VRP CV-1), which serves to emulate the variable water demand by regulating the cross-sectional area through which flows through the pipe.

The electrical signals from the sensors in the form of a current (4–20 mA) are converted to voltage (0–10 V) by an electrical conditioning board. Afterward, the voltage levels are converted into a digital signal by a data acquisition system (DAQ) model NI-USB 6229, with a sampling frequency set at 10 Samples/s.

Finally, the digital signal was transmitting via USB to a personal computer (PC) for storage and digital processing. Next, application of the control algorithm was carried out, and then, an actuation signal was generated and sent from the PC to the frequency inverter via DAQ.

## 4. Background Definitions

In this section, we showed the main concepts of the indirect adaptive control model by the reference model and the correntropy theory as error minimization criteria for the estimation of the adaptive system parameters.

### 4.1. Adaptive Controller

Generally, industrial processes have aspects of nonlinearities, parameter changes, disturbances, impulsive noise, and the influence of outliers in the sensor’s signal measurements. These characteristics indicate the need to adopt more robust controllers, like adaptive controllers, given the criticality and the search for maximum efficiency in industrial processes [26].

The objective of adaptive control is to maintain the system control’s performance, even in the influence of uncertainties or parametric variations in the plant. In general, there are two types of methods for building adaptive controllers: direct and indirect. In the direct method, the controller gains are estimated directly from a pre-established reference model, that is, it is not necessary to perform the identification of the plant parameters [27].

In the indirect method, the plant model is determined as a function of the unknown plant parameter vector, requiring a real-time estimator, using the plant’s input and output signals. Therefore, the generated model is treated as true and its parameters are used for the calculation of the controller variables [27].

For the direct method, most works use the Adaptive Reference Model Controller (MRAC) topology. In this controller, the plant output signal is compared with the reference model output signal, generating a tracking error. The controller parameters are adjusted, using a cost function, based on this error, making the plant output signal converge with that of the reference model. The matching condition is reached when the tracking error is zero [28].

**Figure 3:**
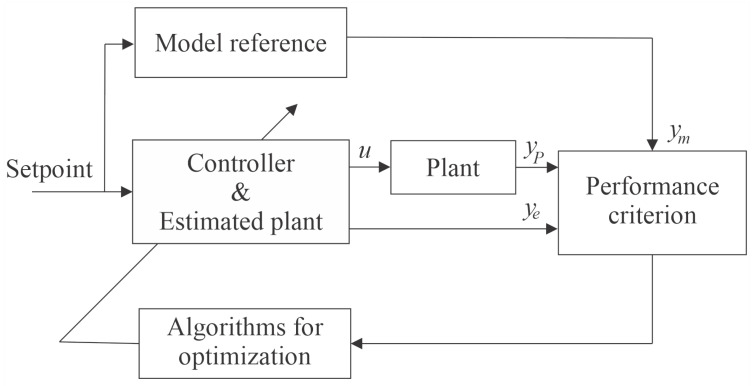
Indirect adaptive control by reference model.

Typically, the parameters of adaptive systems use the MSE as a cost function to compose the performance criteria, which evaluates the error between the actual measured value and the values of the estimated plant and reference model, as shown in the diagram of Figure 3. In this Figure, *u* is the controller signal, $y_{p}$ the plant signal, $y_{e}$ the estimated plant model signal, and $y_{m}$ the reference model signal.

However, the satisfactory performance of the MSE criterion is based on the consideration that probabilistic characteristics of the error, i.e., that the probability density function is Gaussian and has zero mean. Consequently, when this characteristic is not observed, which happens especially when the measured signal is subjected to outliers, this performance criterion distorts the model, decreasing the reliability of system and the adaptation mechanism.

Therefore, it is relevant to study another performance criterion that can naturally reject outliers without the need for pre-processing and maintain the performance of the control system. Thus, in this work, it is proposed to apply the criterion of Maximum Correntropy, whose definitions will be briefly discussed in the next subsection.

### 4.2. Correntropy Theory

Improved strategies should be used for the optimal development of adaptive controllers, whose parameters are determined using mathematical algorithms capable of extracting the maximum possible information contained in the error PDF. For this purpose, the error PDF model, in general, can be characterized by higher-order statistical moments such as Skewness and Kurtosis. Therefore, nonlinear systems can utilize all information contained in the process measurements synergistically, increasing the robustness and performance of the controllers [26].

To satisfy these requirements, in this work, correntropy was used as the minimization criterion, which can be conceptualized as the generalization of the correlation. This is a metric from Information Theory, which measures the generalized similarity between two random variables X and Y, and its mathematical formula is defined in the following expression [12]:

(1) $$\upsilon \left(X,Y\right)=E_{XY}\left[\kappa \left(X-Y\right)\right]$$

where E[.] is the statistic expectation operator, and κ is a gaussian kernel. In this paper, the Gaussian kernel will be adopted, described as

(2) $$\kappa \left(x-x_{k}\right)=\frac{1}{\sigma \sqrt{2\pi }}exp\left(-\frac{{\left(x-x_{k}\right)}^{2}}{2\sigma^{2}}\right)$$

where *x* is the estimated value, $x_{k}$ are the *k* values measured around *x*, and σ is the standard deviation, as described in Equation (2).

In practice, one does not have knowledge of the probability density function, only of a finite number of data $\left\{{{\left(x_{i},y_{i}\right)}_{i=1}}^{N}\right\}$. In this situation, one strategy that can be used to estimate the unknown values is that of the Parzen Windows [12], the mathematical form of which is described by

(3) $$\upsilon_{\sigma ,N}\left(X,Y\right)=\frac{1}{N}\sum_{i=1}^{N}\kappa \left(x_{i}-y_{i}\right)$$

In general, when disregarding the influence of the σ parameter and adopting the Gaussian kernel the correntropy is symmetric, positive, bounded, and results in the weighted sum of all even moments of the random variable *Y*-*X* [12].

In addition, correntropy is related to a distance measure, called the Correntropy Induced Metric (CIM) between two arbitrary scalar random variables *X* and *Y*, as shown in Equation (4), satisfying all the properties of a metric [12].

(4) $$CIM\left(X,Y\right)={\left(\upsilon_{\sigma ,N}\left(0,0\right)-\upsilon_{\sigma ,N}\left(X,Y\right)\right)}^{1/2}$$

CIM divides the space into three different regions: when the error is close to zero, CIM is equivalent to a L2 norm (Euclidean, similar to the MSE criterion); when the error increases, CIM becomes similar to a L1 norm (transient, sum of coordinate differences); when the error is very large, CIM becomes a L0 norm, the metric saturates and becomes very insensitive to large errors, characterizing a rejection region [29].

This characteristic shows the importance of defining the width of the Gaussian kernel, i.e., the smaller this width, the smaller the Euclidean region. On the other hand, increasing the kernel size will also increase the Euclidean region, making the metric behave like the MSE criterion.

A large number of adaptive algorithms use the least squares algorithm (LMS). LMS is a stochastic gradient algorithm on the least mean square error (MSE) criterion, which works well for linear and Gaussian systems. However, its performance will become poor when the signals are not Gaussian, especially when the systems are disturbed by impulsive noise. On the other hand, the MCC excels the MSE criterion because it is a non-linear similarity measure that contains higher order statistics of the signals and is insensitive to large discrepancy values [22].

## 5. Methodology

The proposed methodology is based on the Maximum Correntropy Criterion with Gaussian kernel as mechanisms for adaptation and estimation of an Indirect Adaptive Control by Reference Model, which we will call IMRAC-PID-MCC and is described in the following.

### 5.1. Controller Structure

**Figure 4:**
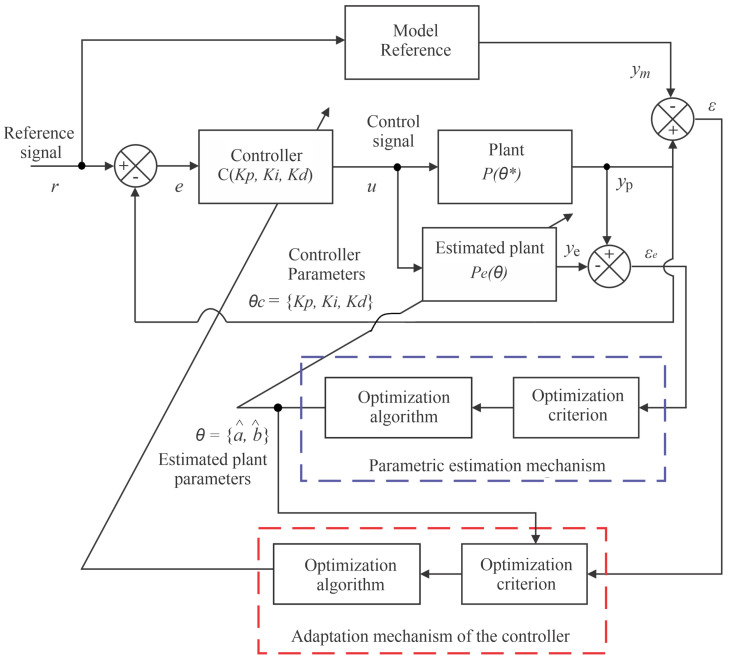
IMRAC-PID block diagram.

Figure 4 shows the block diagram of the proposed control system, consisting of 6 subsystems:

- Reference Model: transfer function with the behavior that will be imposed on the controlled system ($H_{p}$);
- Controller: proportional-Integral-Derivative with variable earnings (*C*);
- Plant: real system to be controlled (*P*);
- Estimated Plant: the Identified Function of the System ($P_{e}$);
- Parametric estimation mechanism: implements Estimation of plant parameters (θ);
- Adaptation mechanism of the controller: update controller’s gains ($\theta_{c}$);

In this control scheme, the plant model $P(\Theta^{*})$ is calculated as a function of the unknown parameter vector $\Theta^{*}$. A real-time estimator generates an estimate θ(t) of $\theta^{*}$ at each instant time *t*, processing the input *u* and the output $y_{p}$. The estimation of the parameters θ(t) will determine an estimated model, characterized by $P^{*}\left(\theta \left(t\right)\right)$ which, for the purposes of the controller design, is treated as the true model of the plant at time *t* and is used to calculate the controller parameters or gain vector **$\theta_{c}\left(t\right)$** through the algebraic equation $\theta_{c}\left(t\right)$ = $F(\theta_{c}\left(t\right))$ [27].

Parametric estimation and adaptation mechanisms can assume different optimization criterion, such as MSE or MCC. In this work, we seek to contrast these two criteria in both mechanisms.

### 5.2. Controller System

For the mathematical development of the proposed control scheme, a first-order plant, Equation (5), will be considered, approximates many water pumping systems, the focus of this paper.

(5) $$H_{p}\left(s\right)=\frac{Y_{p}\left(s\right)}{U(s)}=\frac{b_{p}}{s+a_{p}}$$

where $H_{p}\left(s\right)$ is the plant transfer function, $Y_{p}\left(s\right)$ is the plant output, U(s) is the plant input, $b_{p}$ and $a_{p}$ are the plant parameters.

The definition of the order of the reference model depends on the order obtained from the closed-loop transfer function of the plant with the controller, which, in this case, yields a second order system. The transfer function of the reference model defines the characteristics of the response that the plant must follow, and mits transfer function $H_{m}\left(s\right)$ is given by

(6) $$H_{m}\left(s\right)=\frac{Y_{m}\left(s\right)}{R(s)}=\frac{b_{m}}{s^{2}+a_{m1}s+a_{m2}}$$

where R(s) is the reference, $Y_{m}\left(s\right)$ is the output of the reference model.

It is important to observe that the order of the plant, in many cases, is unknown and can be obtained through a test or empirically defined by the designer and influences the performance of the controller, as the controller parameters must be adjusted for the plant to converge to the reference model.

Finally, a Proportional Integral Derivative (PID) controller is adopted, whose parameters to be adapted are $K_{p}$, $K_{i}$ and $K_{d}$, respectively, with transfer function $H_{c}\left(s\right)$ given by

(7) $$H_{c}\left(s\right)=K_{p}+\frac{K_{i}}{s}+sK_{d}$$

The closed loop equation is then given by

(8) $$y_{p}\left(t\right)=\frac{{\left(\frac{d(.)}{dt}\right)}^{2}\left(K_{d}b_{p}\right)+\left(\frac{d(.)}{dt}\right)\left(K_{p}b_{p}\right)+K_{i}b_{p}}{{\left(\frac{d(.)}{dt}\right)}^{2}\left(1+K_{d}b_{p}\right)+\left(\frac{d(.)}{dt}\right)\left(a_{p}+K_{p}b_{p}\right)+K_{i}b_{p}}r\left(t\right)$$

To approximate the closed-loop output signal of the plant ($y_{p}$) to the reference model output signal ($y_{m}$), it is necessary to minimize the tracking error ($\varepsilon =y_{p}-y_{m}$). This error will be minimized by maximizing the similarity between the plant signal and the reference model using the Maximum Correntropy criterion, whose cost function is given by:

(9) $$J_{MCC}=\frac{1}{\sigma N\sqrt{2\pi }}\sum_{n=1}^{N}exp\left(-\frac{{\left({y_{p}}_{n}-{y_{m}}_{n}\right)}^{2}}{2\sigma^{2}}\right)$$

where *N* is the observation window, that is, the number of measured values, and σ is the width of the Gaussian kernel (standard deviation).

To maximize the value of $J_{MCC}$, it is reasonable to modify the parameters $K_{p}$, $K_{i}$, and $K_{d}$ toward their ascending gradient. Replacing (8) in (9) and knowing that the partial derivative of $y_{m}$ is equal to zero because this does not depend on the parameters to be adapted, D=d(.)/dt and considering N=1, one obtains

(10) $$\frac{dK_{p}}{dt}=\Gamma \left(t\right)\left(\frac{\left(\frac{b_{p}}{1+K_{b}b_{p}}\right)D}{D^{2}+D\left(\frac{a_{p}+K_{p}b_{p}}{1+K_{d}b_{p}}\right)+\left(\frac{K_{i}b_{p}}{1+K_{d}b_{p}}\right)}\right)e\left(t\right)$$

(11) $$\frac{dK_{i}}{dt}=\Gamma \left(t\right)\left(\frac{\left(\frac{b_{p}}{1+K_{b}b_{p}}\right)}{D^{2}+D\left(\frac{a_{p}+K_{p}b_{p}}{1+K_{d}b_{p}}\right)+\left(\frac{K_{i}b_{p}}{1+K_{d}b_{p}}\right)}\right)e\left(t\right)$$

(12) $$\frac{dK_{d}}{dt}=\Gamma \left(t\right)\left(\frac{\left(\frac{b_{p}}{1+K_{b}b_{p}}\right)D^{2}}{D^{2}+D\left(\frac{a_{p}+K_{p}b_{p}}{1+K_{d}b_{p}}\right)+\left(\frac{K_{i}b_{p}}{1+K_{d}b_{p}}\right)}\right)e\left(t\right)$$

where

(13) $$\Gamma \left(t\right)=-\frac{1}{\sigma^{3}\sqrt{2\pi }}exp\left(-\frac{{\varepsilon (t)}^{2}}{2\sigma^{2}}\right)\varepsilon \left(t\right)$$

Equation (13) provides the controller adaptation mechanism with a natural rejection of spurious values or outliers due to the quadratic exponential decrease of the tracking error, ε(t).

### 5.3. System Identification Mechanism

The adopted model of the estimated plant can be written as is the first order Autoregressive-Exogenous (ARX) equation, defined by

(14) $$y_{e}\left[k\right]=a_{1}u\left[k\right]+a_{2}u\left[k-1\right]+a_{3}y_{e}\left[k-1\right]$$

where $a_{1}$, $a_{2}$, and $a_{3}$ are the estimated plant parameters; *u* is the excitation signal; and $y_{e}$ is the estimated output. The estimated parameters are updated using Equations (15)–(17).

(15) $$a_{1}\left[k\right]=a_{1}\left[k-1\right]+\Gamma_{e}\left[k\right]u\left[k\right]$$

(16) $$a_{2}\left[k\right]=a_{2}\left[k-1\right]+\Gamma_{e}\left[k\right]u\left[k-1\right]$$

(17) $$a_{3}\left[k\right]=a_{3}\left[k-1\right]+\Gamma_{e}\left[k\right]y_{e}\left[k-1\right]$$

where

(18) $$\Gamma_{e}\left[k\right]=-\frac{1}{N_{e}{\sigma_{e}}^{3}\sqrt{2\pi }}\sum_{k=1}^{N_{e}}exp\left(-\frac{{\varepsilon_{e}\left[k\right]}^{2}}{2{\sigma_{e}}^{2}}\right)\varepsilon_{e}\left[k\right]$$

Applying Tustin’s discretization method to (5), the discrete transfer function of the plant, with sampling period *T*, is obtained, as shown in (19). This discretization method guarantees the stability of the system on every unit circle in the *Z* plane [30].

(19) $$y_{p}\left[k\right]=\left(\frac{\left(a_{p}T\right)\left(u\left[k\right]+u\left[k-1\right]\right)}{2+Tb_{p}}\right)+y_{p}\left[k-1\right]$$

Thus, for the estimation error $\varepsilon_{e}$ to be zero, $y_{p}\left[k\right]$ must equal $y_{e}\left[k\right]$, that is, (14) and (19) must be equivalent, obtaining

(20) $$a_{1}=a_{2}=\frac{a_{p}T}{2+Tb_{p}}$$

(21) $$a_{3}=\frac{2+b_{p}T}{2+Tb_{p}}$$

The estimates of ap and bp, represented by ${\hat{a}}_{p}$ and ${\hat{b}}_{p}$ can be found by solving the system of equations given by (20) and (21).

(22) $${\hat{a}}_{p}=\frac{2a_{1}+a_{1}b_{p}T}{T}$$

(23) $${\hat{b}}_{p}=\frac{2a_{3}+2}{T-a_{3}T}$$

Given that, since ${\hat{a}}_{p}\to a_{p}$ and ${\hat{b}}_{p}\to b_{p}$, the mathematical formulation for estimating the controller parameters, presented in Equations (10)–(12), should use the estimated values of the parameters instead of the real ones. The adaptation mechanism laws of $K_{p}$, $K_{i}$, and $K_{d}$ ensure that the plant response converges asymptotically to the reference model response for any value of the reference signal, r(t). This statement is plausible, since $J_{MCC}\left(\varepsilon \right)>0$, ${\dot{J}}_{MCC}<0$, meeting Lyapunov stability criterions.

However, when ||ε||→∞, $J_{MCC}\left(\varepsilon \right)\to 0$, $J_{MCC}\left(\varepsilon \right)\to 0$, this, according to [16], determines that ||ε||=0 is not an asymptotically globally stable point, because for it to be, it would require ||ε||→∞ and $J_{MCC}\left(\varepsilon \right)\to \infty$.

## 6. Experimental Results

In this section, the performance of the adaptive control algorithm IMRAC-PID-MCC is evaluated through experimental tests, whose objective is to identify and control the pressure measured by the PT-3 sensor, as illustrated in Figure 2. To achieve these objectives, the proposed experimental scenarios are (i) definition of the reference model; (ii) identification of the system based on MCC; (iii) adaptive PID control based on MCC; (iv) analysis of the controller performance with changing demand; (v) performance evaluation of the system in the presence of outliers; and (vi) comparison of the proposed method based on MCC concerning the classical technique, MSE.

In addition, the speed of rotation of the motor pump was regulated through the use of a frequency converter. The drive, sensor data acquisition, and implementation of the adaptive controller took place in a virtual environment developed in LabVIEW software.

### 6.1. Model Reference Definitions

**Figure 5:**
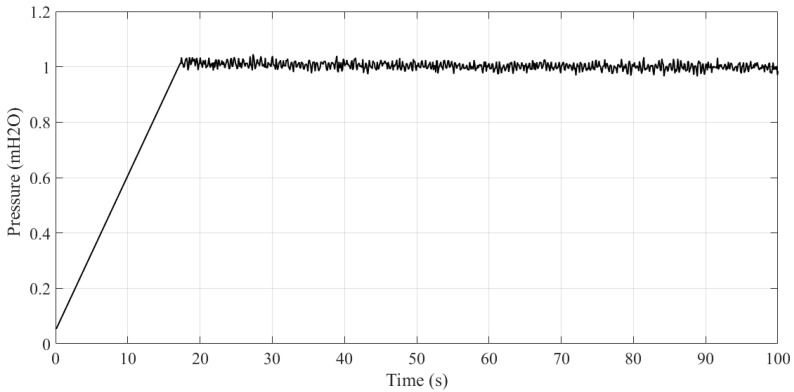
Normalized unit step response of the open loop plant.

To build the reference model, it is first necessary characterize the plant to be controlled. For this reason, the normalized step response of the plant was obtained, as illustrated in Figure 5. It can be observed that the system takes 17 s to reach its maximum value, which corresponds to the acceleration ramp of the frequency inverter, whose inferiorly limits the characteristics of the reference model.

Given the temporal characteristics obtained in Figure 5 and knowing that this is a water supply system, it was empirically defined that the reference model should present the smooth transient regime, to avoid abrupt transients and the hydraulic phenomenon called water Hammer, which causes an abrupt hydraulic transient and may cause rupture in the ducts.

For the reference model, the second-order transfer function expressed by Equation (24) was adopted. The values of the damping frequency ($w_{n}$), equal to 1.41, and damping factor (ξ), equal to 3.54, were determined empirically on observing the system response. The purpose of adopting these values is to give the system a smooth transient with the same settling time as the step response of the real system used in the experiments.

(24) $$H_{m}\left(s\right)=\frac{Y_{m}\left(s\right)}{R(s)}=\frac{{w_{n}}^{2}}{s^{2}+2\xi w_{n}+{w_{n}}^{2}}=\frac{2}{s^{2}+10s+2}$$

**Figure 6:**
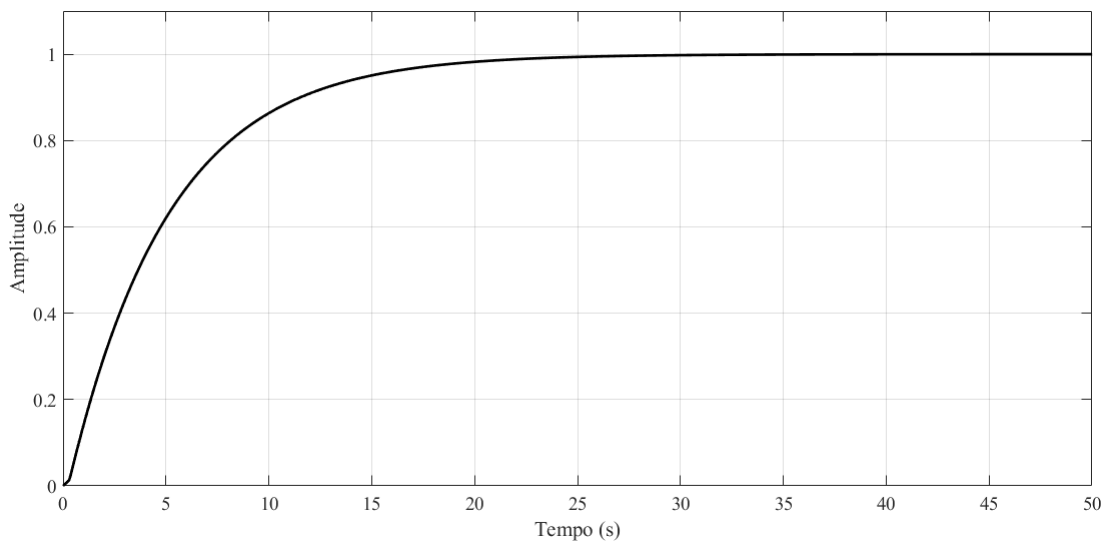
Response to the unit step of the reference model.

Figure 6 shows the step response of the reference model.

### 6.2. System Identification Based on MCC

For the system identification using (22) and (23), the effect of the observation window and Gaussian kernel width on plant identification performance is evaluated.

The premises considered were $K_{p}$, $K_{i}$, $K_{d}$, $a_{1}$, $a_{2}$, and $a_{3}$ equal to 0.01; $N_{e}$ equal to 5; $\sigma_{e}$ equal to 15; sampling period *T* equal to 0.1 s; and the desired pressure value equal to 10 m H${}_{2}$O. The duration interval of each experiment was 180 s.

**Figure 7:**
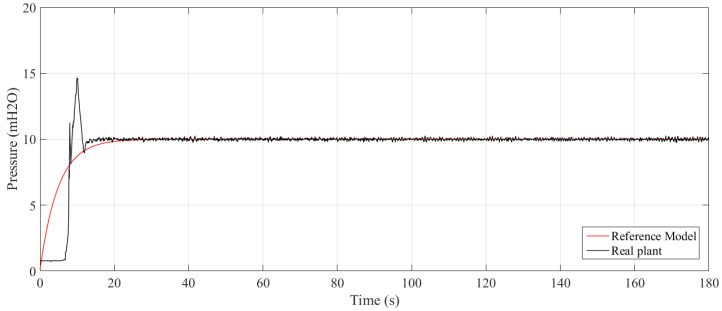
Step response to N=1 and σ=10.

**Figure 8:**
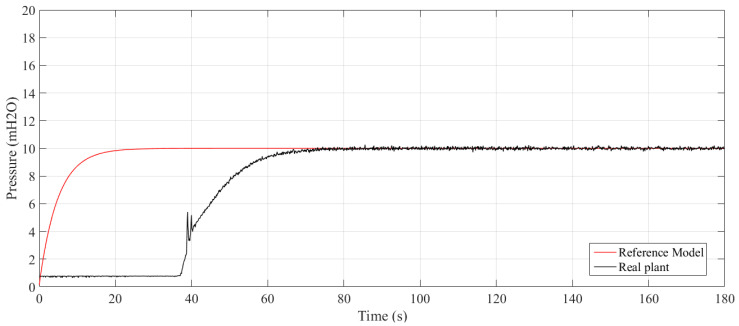
Step response to N=20 and σ=2.

There were 20 experiments conducted varying the value of sigma and N. The dynamic results are summarized in Table 1, namely, rise time ($t_{r}$), settling time ($t_{s}$), peak time ($t_{p}$), delay time ($t_{d}$), percentage overshoot ($M_{p}$), and percentage mean error ($\bar{E}\left(\%\right)$). Figure 7 and Figure 8 show the controlled pressure temporal response two of those experiments.

The values of the system transient response shown in Table 1 are more sensitive to the width of the Gaussian kernel σ than to the observation window width *N*, which is due to the exponential decay present in the cost function. Therefore, the smaller the size of the windows, the greater the weight assigned to the statistical moments, leading to faster responses and the emergence of percentage overshoot. On the other hand, increasing values of *N* and σ cause an increase in rising time, settling time, peak time, and the mitigation of the overshoot.

Moreover, there is no significant impact on the error in the permanent regime, due to the integrative action of the controller. When dealing with a water system, the adoption of N and σ values that cause the appearance of overshoot and abrupt transient response is not appropriate, as they can cause damage to the pipes.

To evaluate the performance of the controller, the correntropy percentage was calculated as

(25) $$\upsilon_{\sigma }\left(\%\right)=exp\left(-\frac{{\left(y_{p}-y_{e}\right)}^{2}}{2\sigma^{2}}\right)\cdot 100\%$$

where $y_{p}$ is the plant output signal, $y_{e}$ is the model output signal, and σ is the kernel width adopted was equal to 1.

This expression allows for a generalized evaluation of the similarity between two variables, which is not the case for methods using Mean Absolute Percentage Error (MAPE) and Integral Square Error (ISE), which are restricted to the quadratic error.

The results obtained in this experiment showed that, in all cases, the value of the correntropy percentage was over 90%, with the maximum value equal to 95.45%, for N=1 and σ=2, which is a consequence of the fast transient response and error in the permanent regime nearing zero.

### 6.3. Robust Adaptive Control Based on MCC

In this experiment, the robustness of the controller to the change in the setpoints values was evaluated. For this, the following was considered: $K_{p}$, $K_{i}$, $K_{d}$, $a_{1}$, $a_{2}$, and $a_{3}$ equal to 0.01; *N* equal to 10; σ equal to 15; sampling period *T* equal to 0.1 s; and the desired values equal to 10, 12, 14, 16, 14, 12, and 10 m H${}_{2}$O. The time interval at each time step was 180 s.

**Figure 9:**
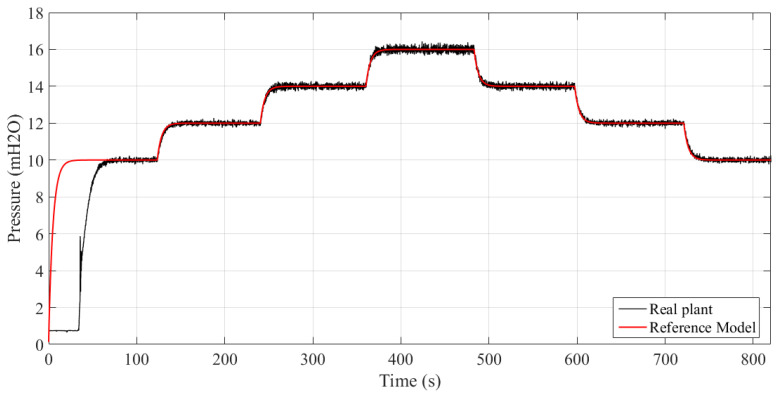
System responses for different setpoints.

Figure 9 shows the system response for different reference values. It is observed that due to the inertia of the pumping system and the tuning performed in the controller, the plant needs 70 s to reach the value of the reference model. This gives the system a soft response with asymptotic convergence.

**Figure 10:**
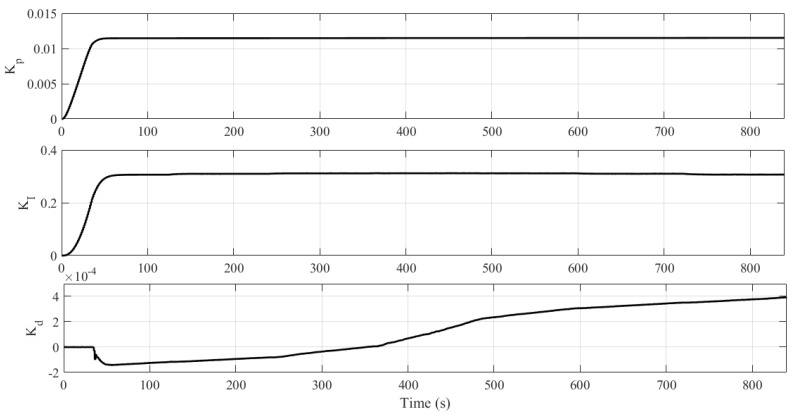
Controller parameters (Experiment II).

Figure 10 shows the variation of the controller parameters with time. It can be seen that, after inertia and initial learning, there is a considerable increase in the convergence speed of the parameters. However, the derivative gain ($K_{d}$) continues to change, maintaining the system steady and with tracking error close to zero, despite the noises in the measured signal.

### 6.4. Variable Demand Pump System

The demand of the pumping system is variable, i.e., there are times with more or less consumption, consequently having variations in pressure in the network throughout the day. This way, the control system must work to maintain the system with a steady and permanent pressure despite these variations.

**Figure 11:**
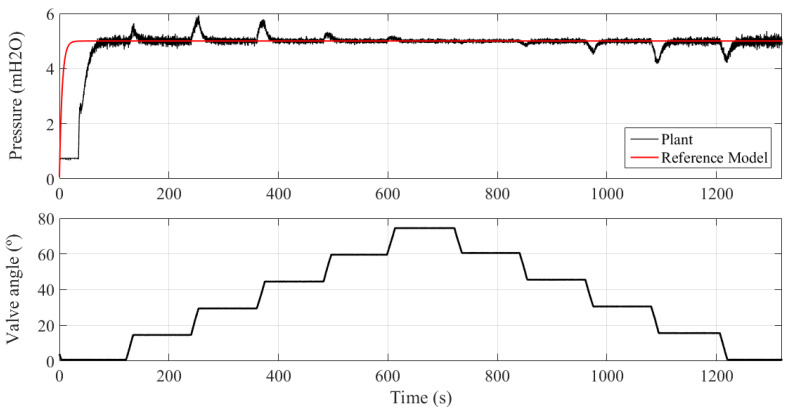
System response under different angles of the VRP CV-1.

Figure 11 shows the operation of the controlled plant when there are variations in water demand, emulated by maneuvering the VRP CV-1 control valve, as shown in Figure 2. The closing of VRP CV-1 causes an increase of the system pressure, so the adaptation mechanism operates to update the controller parameters so that the plant signal converges to the reference model.

On the other hand, the opening of VRP CV-1 causes a reduction in pressure, with the adaptation mechanism also actuating. In these two situations, during the transition, the maximum error was 15.8%.

**Figure 12:**
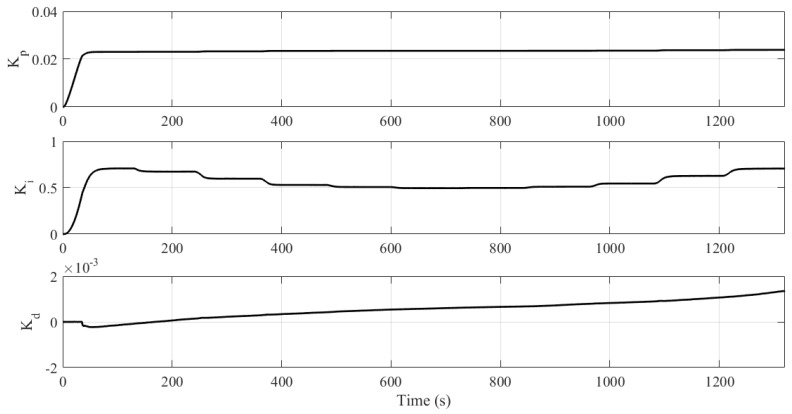
Evolution of controller parameters (Experiment III).

Concerning the controller parameters, it is noted that $K_{p}$ remains constant since the error during the transient is around zero, but $K_{i}$ and $K_{d}$ are changed so that the system, by changing the setpoint, asymptotically converges to zero error in the steady state, as illustrated in Figure 12.

### 6.5. Performance Evaluation of System with Outliers

In this experiment, the robustness of the controller to outliers are investigated, which can be frequently associated with communication failures or physical damage to the transducers. The premises adopted for this experiment are the same as those used in the previous experiment. The total test time was 4 min, and the outliers started after 2 min of operation, i.e., when the system was in a permanent regime.

**Figure 13:**
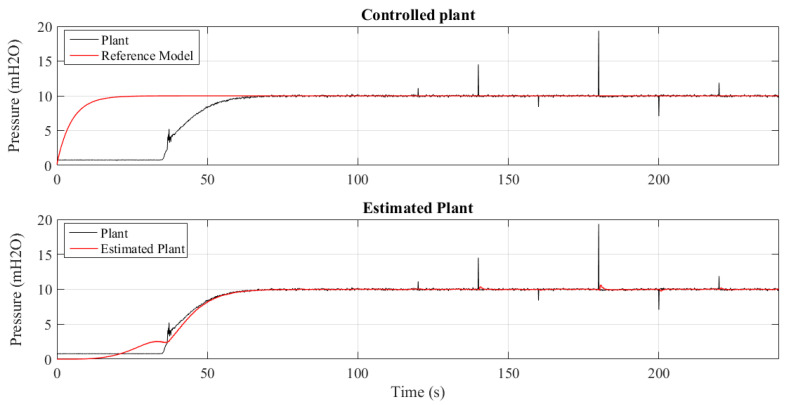
System controlled using MCC criterion subjected to outliers every 20 s.

**Figure 14:**
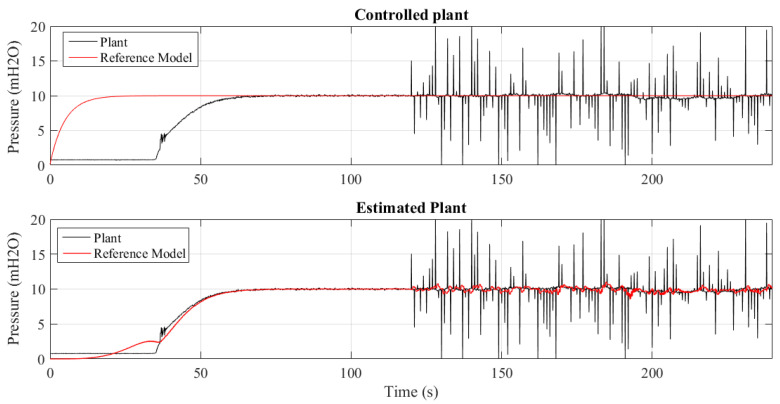
System controlled using MCC criterion subjected to outliers every 1 s.

Figure 13 and Figure 14 show 2 results of the controlled pressure out of the 4 experiments performed for different insertion periods of the outliers: 20, 10, 5 and 1 s. It can be seen that the control mechanism and parametric estimation act to be effective in maintaining the system pressure stability without deviating from the value of the reference model. This shows the tolerance of the MCC to the occurrence of outliers.

The quantification of the of parametric tracking and estimation errors in permanent regime are presented in Table 2 and Table 3, respectively. It can be seen, for the tracking error, that the reduction of the outlier insertion period causes the symmetry of the system, quantified by Skewness, increasing the histogram flattening and width. However, it maintains the average error close to zero.

The identification process ignores the nonlinearities imposed by the introduction of outliers. Since the controller signal depends on the identified plant signal, this robustness is essential for the control signal to remain constant, as quantified by the statistical measurements.

**Figure 15:**
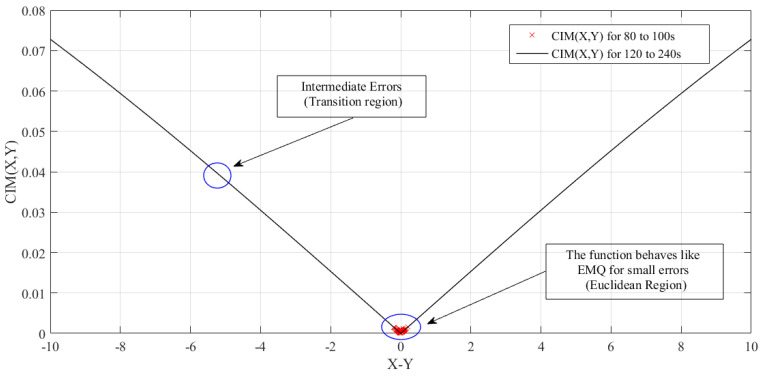
Measurement of the CIM for parametric estimation error.

Another way to see the rejection of outliers is through the Correntropy Induced Metric (CIM), as shown in Figure 15. Consider the worst case scenario, where the noise insertion time interval is 1 s, as shown in Figure 14 and also consider a Equation (26), where the desired value is X={10} and the values assured by the PT-3 transducer are described by *Y*. In addition, N=10 and σ=15 were adopted in this calculation, which corresponds to the tuning values of the controller’s adaptation mechanism, resulting in

(26) $$CIM\left(10,Y\right)=0.16\cdot {\left(1-exp\left(-\frac{{\left(10-Y\right)}^{2}}{2\cdot {\left(0.027\right)}^{2}}\right)\right)}^{1/2}$$

### 6.6. Performance Comparison between MCC and MSE Criterion

In the current experiment, to compare the use of the proposed criterion, the same previous experiment was performed (Section 6.5) but now using as criterion the Mean Squared Error, described by Equation (27), to compose the parametric estimation mechanism and controller adaptation.

(27) $$J_{MSE}=\frac{1}{N}\sum_{n=1}^{N}\frac{1}{2}\varepsilon^{2}$$

where *N* is the observation window, and ε is the error between the measured and estimated value.

Using $\varepsilon =y_{p}-y_{m}$ in Equation (27), where $y_{p}$ is defined by Equation (5), and $y_{m}$ is considered constant, since it does not depend on the parameters to be adjusted. Therefore, to minimize the value of $J_{MSE}$, one must modify the parameters $K_{p}$, $K_{i}$, and $K_{d}$ in direction of their downward gradient, such as

(28) $$\frac{dK_{p}}{dt}=-\gamma_{p}\frac{\partial J_{MSE}}{\partial K_{p}}$$

(29) $$\frac{dK_{i}}{dt}=-\gamma_{i}\frac{\partial J_{MSE}}{\partial K_{i}}$$

(30) $$\frac{dK_{d}}{dt}=-\gamma_{d}\frac{\partial J_{MSE}}{\partial K_{d}}$$

where $\gamma_{p}$, $\gamma_{i}$, and $\gamma_{d}$ are the controller adaptation gains, which adjust the learning rate.

For the parametric estimation mechanism, the same procedures exposed above were performed. For this, $\varepsilon =y_{p}-y_{e}$ will be considered, replacing Equations (14) and (5) in continuous form in Equation (27) and applying the descending gradient method for each of the estimated plant values, obtaining

(31) $$\frac{da_{1}}{dt}=-\gamma_{a_{1}}\frac{\partial J_{MSE}}{\partial a_{1}}$$

(32) $$\frac{da_{2}}{dt}=-\gamma_{a_{2}}\frac{\partial J_{MSE}}{\partial a_{2}}$$

(33) $$\frac{da_{3}}{dt}=-\gamma_{a_{3}}\frac{\partial J_{MSE}}{\partial a_{3}}$$

The parameters of the controller that use the MSE as a criterion were empirically adjusted as $\gamma_{a_{1}}=8\times 10^{-5}$, $\gamma_{a_{2}}=8\times 10^{-5}$, $\gamma_{a_{3}}=4\times 10^{-5}$, $\gamma_{p}=8\times 10^{-5}$, $\gamma_{I}=8\times 10^{-5}$, $\gamma_{d}=4\times 10^{-5}$, $N_{e}=3$ and N=6.

**Figure 16:**
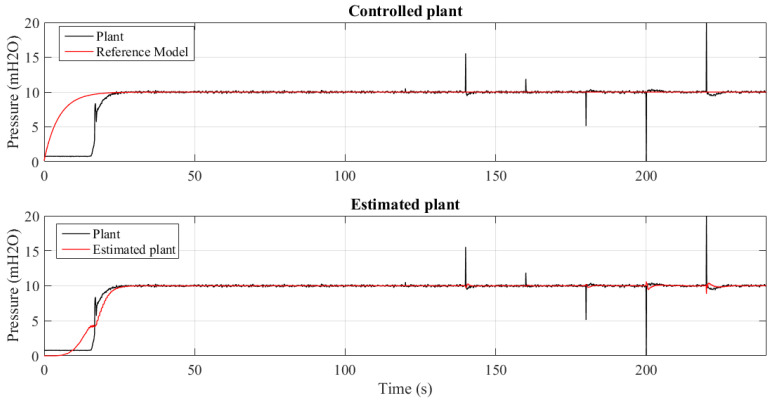
System using the MSE criterion subjected to outliers at intervals of every 20 s.

**Figure 17:**
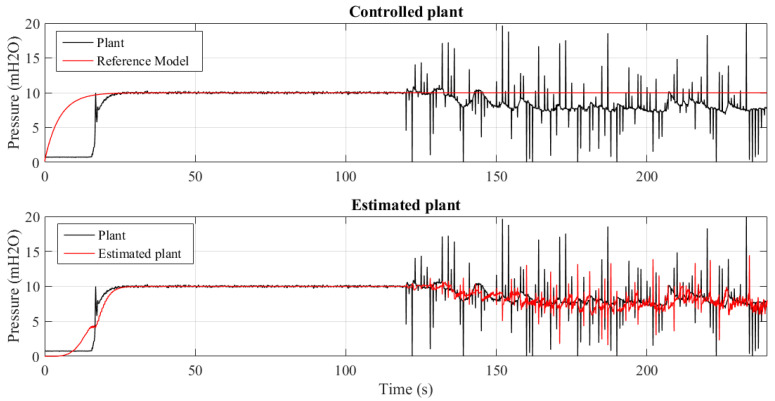
System using the MSE criterion subjected to outliers at intervals of every 1 s.

Figure 16 and Figure 17 show 2 of the 4 trials performed for different insertion periods of the outliers: 20, 10, 5, and 1 s. Comparing these results with those presented in the previous experiment, it can be seen that for long periods of insertion of outliers, the controller using the MSE criterion performs similar to the MCC; due to the reduced amount of outliers, the average error is close to zero, the Euclidean region of the CIM space.

On the other hand, when the period is short and consequently there are more outliers, the MSE criterion is less robust to the presence of outliers than the controller that makes use of the MCC, as can be shown in Figure 14 and Figure 17. This fact happens because the MCC has a natural rejection of outliers, a result of the exponential decay of the Gaussian kernel.

The quantification of the error in the permanent regime, of tracking and parametric estimation, is presented in Table 4 and Table 5, respectively. The intolerance to outliers of the MSE criterion results from its mathematical formula, which only has first and second-order statistical moments, consequently adding to the model the spurious values included in the error.

The exponential decay of the mathematical expression of the Maximum Correntropy Criterion with Gaussian kernel (Equation (9)), naturally rejects the spurious values of the tracking and estimation error, that is, when the error will tend to infinity, as in the case of outliers, this decay assigns a minimum value to the cost function, consequently reducing the impact of these undesired values on the parameters to be adapted.

For the experiments where no outliers are present, both controller error evaluation criteria showed similar characteristics. This is expected because the Maximum Correntropy criterion approximates the MSE criterion when the errors tend to zero. On the other hand, when there is excessive presence of outliers, as in the case of the outlier insertion interval every 1 and 5 s, the use of MCC reduced the average tracking error by 1700% and 31,443%, respectively, compared to the MSE criterion. The estimation error, in this same situation, is reduced by 32,833% and 1800%, respectively.

## 7. Conclusions

In this work, an indirect adaptive control system by reference model was proposed for modeling and controlling hydraulic pressure in water supply systems (IMRAC-PID-MCC). The methodology was developed following the concepts of Information Theory, specifically, the definitions associated with Maximum Correntropy.

The following advantages of using the proposed controller for pressure management in a water supply system are noted:

- It does not require an expert to determine the rules that limit the controller’s action, as in the case of a controller based on Fuzzy logic. Therefore, if the system changes its dynamics, the IMRAC-PID-MCC can adapt to reduce the error to zero;
- It performs system identification and controller parameter updating in real time and does not require a prior data set to perform these functions, as in the case of ANN-based controllers. This is an important advantage, because in many cases, the data modeling the water consumption profile, pumping system output pressure, variation in reservoir level, and the times at which the pump was actuated are not known;
- Because IMRAC-PID-MCC uses a reference model, it provides the knowledge of the mathematical equation that models the system to perform the controller tuning, as in the cases of using the PID controller and Predictive Model.

The adjustment of the kernel width and observation window changes the dynamics of the controlled plant. An excessively small kernel width can cause instability and/or oscillations during the transient regime of the controlled system because it makes it sensitive to error variation. However, excessively large values cause the loss of information, because they approximate the error PDF to a Gaussian, making the information contributions of higher-order statistical moments irrelevant.

The controller proved to be robust to variations in the reference value, changes in the plant characteristics, diverging by a maximum of 15% during the periods of change, in all cases demonstrating percentage overshoot less than 5% and steady-state error less than 2%.

The robustness to outliers allows the system to remain stable in cases where the transducer of the variable to be controlled is damaged, and consequently, there is a failure in the measured signal. In water supply systems, such failures can be caused by electromagnetic interference from the pumping station motors, cable breakdown, or as a result of excessive vibrations.

For future work we propose to investigate the comparison of Indirect Adaptive Control by Reference Model with Direct Adaptive Control by Reference Model, using the same criteria and optimization algorithm. Another recommendation is improving the adaptation mechanism to increase convergence rate and reduce stagnation in local points of minimal.

## Figures and Tables

**Table 1:** Time response characteristics.

*N*	σ	$t_{r}$ (s)	$t_{s}$ (s)	$t_{p}$ (s)	$t_{d}$ (s)	$M_{p}$ (%)	$\bar{E}$ (%)
1	2	8.6	20.5	9.9	7.7	45.5	7.2
	4	10	19.7	11.95	4	47.1	6.2
	6	11	24.5	13.7	13	35.0	6.3
	8	14.6	21	14.58	16	7.0	6.1
	10	28.5	32	-	17	0	5.8
5	2	10.44	33	11.94	11	48.0	6.2
	4	12	20	13.67	12	3.2	6.2
	6	26	40	-	17	0	6.0
	8	39.6	42	-	22	0	5.9
	10	49	47.17	-	27.15	0	5.9
10	2	12	27.33	12.63	10.5	15.6	6.0
	4	29.18	27.7	-	16.68	0	6.1
	6	35.5	40	-	20	0	5.7
	8	48.68	50.5	-	27.58	0	6.4
	10	64.5	63	-	33.4	0	6.1
20	2	25	25	-	14	0	6.0
	4	31.6	32.36	-	19.5	0	5.9
	6	40	45	-	27.19	0	6.3
	8	60	83.56	-	32	0	6.0
	10	76	100	-	39	0	9.0

**Table 2:** Statistical measurements of the rastreaming error of the controlled system using the MCC criterion.

Time Interval between Outliers	Mean	Standard Deviation	Skewness	Kurtosis
20 s	0.0014	0.33	−20.16	557.10
10 s	−0.0020	0.61	−4.41	167.46
5 s	0.0016	0.78	0.49	108.48
1 s	0.0965	1.69	0.17	21.92

**Table 3:** Statistical Measures of plant identification error using the MCC criterion.

Time Interval between Outliers	Mean	Standard Deviation	Skewness	Kurtosis
20 s	0.0026	0.0610	−4.38	39.85
10 s	0.0003	0.1091	−1.13	15.26
5 s	0.0015	0.1385	0.30	9.54
1 s	0.1023	0.3477	−0.12	3.12

**Table 4:** Statistical measurements of the tracking error of the controlled system using the MSE criterion.

Time Interval between Outliers	Mean	Standard Deviation	Skewness	Kurtosis
20 s	0.0013	0.4818	−0.4504	333.44
10 s	−0.0044	0.4484	−2.36	208.69
5 s	0.5031	1.1405	0.02	24.10
1 s	1.6478	1.8276	−0.4295	14.35

**Table 5:** Statistical Measures of plant identification error using the MSE criterion.

Time Interval between Outliers	Mean	Standard Deviation	Skewness	Kurtosis
20 s	0.0029	0.0800	2.82	49.53
10 s	−0.0004	0.0698	−1.1177	32.03
5 s	0.4925	0.7190	1.2010	3.93
1 s	1.8365	1.2868	−0.1600	5.75

## Data Availability

Not applicable.

## Abbreviations

The following abbreviations are used in this manuscript:

ANN	Artificial Neural Network
ARX	Autoregressive-Exogenous
CIM	Correntropy Induced Metric
DAQ	Data Acquisition System
FT	Flow Transducers
GMV	Generalized Minimum Variance
ILC	Interactive Learning Control
IMRAC-PID-MCC	Indirect Adaptive Control by Reference Model based on the Maximum
	Correntropy Criterion
ISE	Integral Square Error
LENHS	Laboratory of Energy Efficiency and Hydraulics in Sanitation
MCC	Maximum Correntropy Criterion
MRAC	Reference Model Adaptive Control
MSE	Mean Squared Error
NLP	Neuro-Linguistic Programming
PC	Personal Computer
PDF	Probability Density Function
PID	Proportional Integral Derivative
PT	pressure transducers
PVC	Polyvinyl chloride
RTC	Real-Time Control
UFPB	Federal University of Paraiba
VRP CV-1	automated proportional valve
